# Supplementary material for: 11-Year Trend in Antibiotic Consumption in a South-Eastern European Country; the Situation in Albania and the Implications for the Future
Source: Antibiotics (Basel). 2023 May 9;12(5):882. doi: 10.3390/antibiotics12050882 (PMC10215466; doi:10.3390/antibiotics12050882)
Supplement: Supplementary file 1 [file antibiotics-12-00882-s001.zip › antibiotics-2349945-supplementary.pdf]

Supplementary Materials

Table S1. - Programs instigated among a selection of LMICs to improve antibiotic prescribing for surgical antibiotic prophylaxis (SAP) patients

Country, year and reference	Summary of the intervention	Outcome
Study involving 4 African countries (Kenya, Uganda, Zambia, and Zimbabwe) 2018 [78]	• The study involved appreciable educational activities – this included five planned visits to each hospital alongside a range of tools to improve SAP<br>• Local teams in the various hospitals identified key areas to concentrate on to improve SAP and identified appropriate indicators<br>• Subsequent monitoring of activities and feedback to further improve SAP	• SSIs decreased to 3.8% of operations post interventions (down from 8.0% - p <0·0001)<br>• Appropriate use of SAP improved to 39.1% of patients – up from 12.8% at baseline (p <0·0001)
Egypt – 2015 [79]	• Extensive education including a 2-day training course coupled with posters and on-the-job training<br>• Regular audit and feedback meetings	• All hospitals showed a significant rise in the optimal duration of antibiotics for SAP (p < 0.01)<br>• The optimal timing of the first dose also improved significantly in 3 hospitals to 38.7% of patients (up from 6.7% p < 0.01)
India - 2018 [80]	• Agreement among key stakeholders regarding the key elements of the WHO checklist to help reduce SSIs<br>• The designated checklist coordinator confirmed that the surgical team had completed its tasks before proceeding to the next steps during the operations	• Prior to implementation - all patients operated on a particular day were administered antibiotics in the morning irrespective of the timing of their surgery<br>• Following the intervention - the correct practice of administration within 1 to 2 hours of the incision became normal practice<br>• In addition, addressing concerns that appropriate surgical patients were not given SAP
Iran - 2019 [81]	• SAP guidelines revised following meetings between pharmacists and the surgical department following lectures and discussion concerning SAP to key members of the surgical departments<br>• Clinical pharmacists participating in ward rounds, communicating with surgeons when guidelines not followed and providing educational material on SAP<br>• Rationality of SAP continually evaluated in accordance with agreed guidelines with clinical pharmacists communicated any concerns directly with relevant physicians	• Rate of antibiotic prescribing beyond 48 hours decreased from 92.1% of patients to 5.7% of patients post intervention<br>• The appropriateness of antibiotic use increased from 30.1% to 91.4% of patients<br>• The mean cost of antibiotics decreased more than 11-fold and length of stay from an average of 5.14 days to 4.33 days (p<0.001)
Kenya - 2013 [82]	• Appreciable educational activities including developing an SAP guideline<br>• Subsequently monitoring adherence to agreed guidelines	• Significant improvement in reducing post-operative antibiotics for SAP to 60% of patients during week 1 and 90% in week 6 post intervention (p< 0.0001)

		• Net reduction in costs including antibiotics and associated consumables
Kenya - 2015 [83]	• Education, training and leadership programs among key front-line staff<br>• Frequent monitoring of activities post-ASP intervention	• SAP patients prescribed antibiotics post-operatively decreased from 50% to 26% of patients<br>• Crude SSI rates significantly decreased to 5% of patients (down from 9.3%)
Moldova - 2015 [84]	• Introduction of a surgical safety checklists in the operating theatre<br>• The data collection team developed and randomly assigned to observe 30% of the surgical cases, appraise adherence to agreed practices as well as feedback the findings	• 12.7% increase in the appropriate use of antibiotics for SAP
Nigeria - 2019 [85]	• Extensive educational activities including development and dissemination of agreed SAP protocol combined with regular meetings with key clinicians and reminders on all-mounted posters<br>• Regular audit and feedback meetings	• SAP patients in the post-intervention period were 5.6 times more likely to receive antibiotics within 60 minutes before the incision vs. pre-intervention (p <0.001)<br>• Redundant antibiotic prescriptions were reduced by 19.1%
Pakistan – 2019 [86]	• Educational interventions including sharing the findings in the pre-intervention arm with all key stakeholders alongside discussions on appropriate SAP<br>• Educational training continued for 10–15 days<br>• Post-intervention - data collected and shared to improve future SAP	• Compliance of correct antibiotic choice, dose, frequency and duration increased from 1.3% to 12.4%<br>• Significant reduction in the mean duration of SAP (17%, p = 0.003), average number of prescribed antibiotics (9.1%, p = 0.014) and average antibiotic cost (25.7%, p = 0.03)<br>• Reduction in hospital costs (p = 0.003) and length of stay (p = 0.023).
South Africa - 2017 [87]	• SAP 'toolkit' alongside regional training and institutional workshops<br>• Consensus and endorsement from key groups for SAP guidelines and modifying guidelines where appropriate<br>• Compliance to SAP guidelines measured alongside feedback to improve future compliance	• Timely administration of antibiotics for SAP increased to 56.4% of surgical patients (P < 0.0001)<br>• Optimal duration of antibiotic administration for SAP increased to 93.9% of patients
Turkey – 2014 [88]	• A number of educational activities were instigated including a series of meetings with physicians from each clinic<br>• Alongside this, daily visits from the Infection Control Nurse as well as regular visits (twice per week) from an Infectious Diseases Control Specialist – more if compliance with agreed guidelines was low<br>• Activities and outcomes regularly shared with the physicians	• Prescribing of appropriate antibiotics for SAP increased from 51% to 63.4% of cases<br>• Duration of prescribing improved from 10.3% to 59.4% of cases<br>• Total cost of antibiotics in surgical units decreased by 38.6%
Turkey – 2019 [89]	Local guidelines updated by two members of the infection control committee with one surgery team leader responsible for improving SAP as part of ASPs in their group	• Compliance with the indication of SAP increased from 55.6% to 64.5% of patients (p<0.05)<br>• Significant impact on antibiotic prophylaxis beyond 24 hours – reducing from 60.2% of

	• Periodic training sessions - supervised and regulated by the SAP surgical team leaders<br>• Agreed that clean and clean-contaminated cases would not be given SAP for longer than 24 hours and that discharge prescriptions would not include antibiotics	patients before the intervention to 7.5% after (p<0.05)<br>• Extent of antibiotic prescribing after discharge reduced from 80.6% of patients to 9.4% (p<0.05)<br>• Limited impact on the timing of the first antibiotic dose – appropriate timing increased from 81.9% of patients to 83.7%

NB: SAP = Surgical antibiotic prophylaxis; SSIs = Surgical Site Infections

Table S2: Initiatives to improve antibiotic utilization among physicians and pharmacists in ambulatory care in LMICs

Country, Year and Reference	Intervention and impact	Impact
**Physicians**		
Malaysia, 2006 [90]	• Principally education<br>• This involved academic detailing from the resident family medicine specialist accompanied by an information leaflet	• Reduction in general antibiotic prescribing rates from 14.3% pre-intervention to 11.0% post-intervention (RR 0.77, 95% CI 0.72 to 0.83)<br>• Reduction in URTI-specific antibiotic prescribing rates from 27.7% and 16.6% post-intervention (RR 0.60, 95% CI 0.54 to 0.66).
Sudan, 2006 [91]	• 20 health centers in Khartoum State were randomly assigned to receive either (a) no intervention; (b) audit and feedback; (c) audit and feedback + seminar; or (d) audit and feedback + academic detailing<br>• The targeted interventions involved audit and feedback alongside academic detailing	• Significant reduction in the mean number of physician encounters with an antibiotic prescribed by 6.3 and 7.7 (p<0.001) at 1 and 3 months post-intervention, respectively<br>• The mean number of encounters where antibiotics were inappropriately prescribed were also significantly reduced post intervention (p<0.001)
Bangladesh, 2007 [92]	• 3-arm study principally involving education divided into 3 groups:<br>• Group I – education surrounding current STGs and subsequent auditing of the prescribing of ARIs<br>• Group-II - Just education surrounding STGs<br>• Group-III - Control group, i.e., no intervention)	• The prescribing of unnecessary antibiotics to treat ARIs was significantly reduced (p<0.01) compared to the pre-intervention period in Group I<br>• The average reduction in antibiotic use (patient encounters) was 23.7% in Group I vs. 15.2% in Group II<br>• When factoring in the Control group (Group III), there was a 15.2% reduction in antibiotic use in Group I as well as 6.9% in Group II
Nepal, 2009 [93]	Principally Education. This included:<br>• Supervision/ monitoring involving periodic visits by district supervisors to 41 PHC facilities<br>• Small-group training among prescribers followed by peer-group discussions alongside self-assessment of the data presented	• In children under five, there was a significant improvement in decreasing the use of antimicrobials to treat children with diarrhea<br>• There was also a significant improvement in the prescribing of antibiotics for patients with URTIs without pneumonia

China, 2014 [94]	• A randomized study to evaluate the effects of capitation with pay-for-performance on antibiotic prescribing, health spending, outpatient visit volume, and patient satisfaction	• Approximately 15% reduction in antibiotic prescriptions<br>• Small reduction in total spending per visit
Thailand, 2014 [95]	Principally education including:<br>• Training HCPs on the rational use of antibiotics<br>• Introduction of practice guidelines<br>• Potential for throat swabs (stool cultures for acute diarrhea)<br>• Printed brochures for patients/ relatives in waiting rooms including the potential harm for antibiotics for URTIs and acute diarrhea	The multifaceted program resulted in:<br>• Limited prescribing of antibiotics for upper respiratory tract infections (13.0%) and for acute diarrhea (19.1%)<br>• Clinical responses on day 3 after receiving care revealed more than 97% of the patients who received antibiotics/ those who did not receive antibiotics were cured or improved
Kenya, 2017 [96]	A comprehensive set of interventions to improve the management of URTIs, UTIs, STIs and childhood diarrhea. These included:<br>• Online educational programs explaining the clinical guidelines for these 4 conditions<br>• 2-hour educational sessions<br>• Monthly feedback meetings<br>• Materials including posters and other material to remind prescribers	• Adherence to agreed clinical quality measures (CQM) increased from 41.4% to 77.1% for PHCs that took part in the intervention but dropped slightly from 26.5% to 21.8% in controls over the 6-month study period. This was greatest for UTIs<br>• Adherence to CQMs significantly increased over the 6-month study period for the active intervention group<br>• The interventions were well received by those operating in the PHCs
Kenya, 2017 [97]	• The study involved a series of educational interventions. These included:<br>o A formal introduction to clinical practice guidelines<br>o Peer-to-peer chart reviews<br>o Peer-reviewed literature describing local AMR patterns<br>o Instigation of quality indicators (QIs) to assess the level of improved prescribing<br>• Five clinical QIs were used with the primary outcome being the proportion of cases in which the guideline-recommended antibiotic was prescribed	• Clinician adherence to the guideline-recommended antibiotics improved significantly during the study period<br>• Adherence increased from 19% of patients at baseline to 68% following all interventions<br>• The composite quality score (secondary outcome) also significantly improved from an average of 2.16 to 3.00 on a five-point scale
India, 2018 [98]	• Principally education to reduce the rate of inappropriate prescribing of antibiotics for URTIs<br>• Initiatives consisted of:<br>o Repeated process of audit and feedback combined with interactive training sessions<br>o One-to-one case-based discussions<br>o STG development and coding updates	• Antibiotic prescribing among 222 audited patients reduced from 62.6% of patients with URTIs prescribed antibiotics to 7.2% following the interventions<br>• This was combined with an increase in the documentation of examination findings from 52.7% to 95.6% of patients justifying the treatment approach
Malaysia, 2019 [99]	Principally education involving educational toolkits included a training module for HCPs on URI and acute diarrhea involving:<br>• One-hour educational sessions covering diagnostic criteria and treatment decision pathways	Appreciable reduction in antibiotic prescribing:<br>• Prescribing for URIs down from 29.1% to 13.7% of patients<br>• Prescribing for acute diarrhea down from 11.2% to 6.7% of patients

	• Educational posters in Malay and English in the waiting area and consultation rooms as well as multimedia educational videos in the waiting areas<br>• Physician reminders	
Namibia, 2020 [100]	• The study assessed the effectiveness of implementation of ASPs among 10 PHCs in Namibia<br>• A SWOT analysis was conducted for each healthcare facility through interviewing infection control focal personnel at each facility – the objective being to assess the level of compliance to good AMS practices and policies	• 90% of the focal persons were aware of systems and polices for good AMS practices<br>• The level of compliance at hospital-based PHCs was 30.8% compared to clinics (9.1%-36.4%)<br>• Principal challenges to implementing ASPs among healthcare facilities in Namibia is a lack of policies and systems specific to antimicrobial use as well as financial and human resource concerns
**Pharmacists**		
Thailand and Vietnam, 2005 [101]	• Multiple interventions and activities including enforcement of regulations banning the selling of antibiotics without a prescription with local inspectors visiting pharmacies to emphasize the importance of prescription-only medicine legislation<br>• In addition, education of pharmacy staff performed face-to-face in Vietnam and by a large group in Bangkok<br>• Alongside this, peer review of pharmacists' activities - voluntary in Thailand and compulsory in Vietnam	• Significant improvements in Vietnam reducing the dispensing of illegal low dose antibiotics (69% vs. 90%), sustained by means of the peer review (71% vs. 95% antibiotics)<br>• In addition, fewer dispensers asked no questions and gave no advice post-intervention (51% vs. 81% for antibiotics)<br>• In Thailand, fewer pharmacists in the group who volunteered for peer review asked no questions and gave no advice for antibiotics requests after peer review (58% vs. 81%)<br>• Currently, limited sanctions (fine of only US$15–25 per documented violation when caught) in Vietnam has resulted in high rates of dispensing of antibiotics without a prescription. Multiple activities, including education of key stakeholders and increasing fines, are needed to reverse the situation [102]
Mexico, 2013 – 2015, [103,104]	• The government in Mexico implemented a number policies in 2010 to enforce existing laws banning the purchasing of antibiotics without a prescription<br>• As part of the policies, antibiotic prescriptions had to be retained and registered in pharmacies<br>• There were fines for non-compliance	• Antibiotic utilization decreased by 22.9% (10.5 to 7.5 DDD/TID) between 2007 and 2012<br>• This trend accelerated after greater enforcement of the legislation<br>• There were significant changes in the dispensing of penicillins and sulfonamides: −0.86 DDD/TID ($p<0.00$) and −0.17 DDD/TID ($p=0.07$) respectively<br>• There was also an appreciable seasonal reduction in the use of penicillins after

		greater enforcement of the legislation in Mexico
Thailand, 2015 [105]	• Principally education involving a multidisciplinary intervention in grocery stores<br>• Trained community leaders were used to reduce the extent of antibiotic availability in these village grocery stores	• 87% fewer antibiotics were available post-intervention compared with pre-intervention<br>• Grocery stores in the control group saw only an 8% reduction in antibiotic availability between the two time periods vs. targeted grocery stores
Republic of Srpska, 2017 [43]	• Education of pharmacists regarding the appropriate management of diseases including ARIs together with the production of guidelines for the 42 most frequent diseases and conditions seen in everyday practice in community pharmacies<br>• Greater enforcement of the regulations of guidelines banning the dispensing of antibiotics without a prescription together with possible fines for violation of laws (Euro500–1500 for pharmacy directors and Euro500–750 for pharmacy technicians)<br>• Special attention given to the importance of adequate communication and skills among pharmacists	• The dispensing of antibiotics without a prescription decreased from 58% to 18.5% of pharmacies<br>• OTC therapy to alleviate symptoms was offered in 72.3% of pharmacies in 2015 up from 67.2% in 2010<br>• OTC medication dispensed included throat and nasal sprays, decongestants, oral expectorants, analgesics and antihistamines<br>• Significantly fewer pharmacies dispensed an antibiotic without a prescription where OTC medicines were offered<br>• Encouragingly, the most common reason for not dispensing to simulated clients was that antibiotics cannot be dispensed without a prescription
Uganda, 2017 [106]	• Four-part intervention to improve the management of paediatric febrile illness among drug sellers:<br>o Training and work activities<br>o Provision of information, education, information and communication<br>o Supply activities including diagnostics and medicines<br>• Monthly support supervision via supervisors trained in either pharmacy or clinical medicine	• The intervention increased the appropriate treatment of children with uncomplicated malaria, pneumonia symptoms and non-bloody diarrhoea by 80.2% (95% CI 53.2–107.2), 65.5% (95% CI 51.6–79.4) and 31.4% (95% CI 1.6–61.2) respectively versus the pre-intervention period<br>• Adherence to guidelines during the intervention phase was high without causing excessive prescribing of antimicrobial medicines
Kenya, 2018 – 2022 [10, 65,66,107-109]	• Education of pharmacists linked to the Pharmacy undergraduate programme at the University of Nairobi (UoN)<br>• Regular interaction with University staff and students regarding key issues surrounding antibiotics and AMR<br>• UoN has also recently launched and implemented an AMS curriculum for undergraduate pharmacy students as part of the NAP	• Low level of dispensing of antibiotics without a prescription (94.1% of antibiotics dispensed with a valid prescription) among pharmacies allied to the UoN<br>• These contrasts with pharmacies not allied to the UoN where 52% of surveyed pharmacists sold antibiotics without a prescription<br>• At the start of the COVID-19 pandemic, there was no purchasing of antimicrobials without a prescription among patients with actual or suspected COVID-19 in community pharmacies allied to the UoN – patients were typically recommended alternative treatments

		• This compares with the study of Kimathi et al. (2022) where 23.4% of respondents had self-medicated with antibiotics during the pandemic, 60.6% at the onset of COVID-19 symptoms before confirmatory tests, with 51.5% self-medicating more than once
China, 2019 [110]	Multiple initiatives were introduced in Shaanxi Province in China to reduce the purchasing of antibiotics without a prescription. These included:<br>• Stricter regulations for dispensing antibiotics<br>• Improving pharmacists' education regarding antibiotics and AMR<br>• Stipulating there must be a qualified pharmacist's present to dispense antibiotics<br>• Increased frequency of unannounced pharmacy inspections<br>• Punishment for pharmacists found to abuse the laws	• The multiple measures resulted in decreased antibiotic sales between 2011 and 2017<br>• There was decreased dispensing of antibiotics without a prescription for a 5-year-old child with diarrhoea between 2011 and 2017 - from 72.3% to 50.2% ($p<0{\cdot}0001$) – simulated patients<br>• There was similar reduction for patients with URTIs – down from 95.8% to 69.5% ($p<0{\cdot}0001$)
Namibia, 2019 – 2021 [67,111,112]	• Education of antibiotics and AMR among pharmacists starting in universities and continuing post qualification<br>• Implementation of regulations banning the purchasing of antibiotics without a prescription<br>• Activities of community pharmacies regularly monitored	• In a survey among 100 households in Namibia, typically cold/flu medication, paracetamol, and decongestants were used to treat adults or their children with ARIs including for common colds and influenza. There was no purchasing of antibiotics without a prescription<br>• There was a similar situation during the COVID-19 pandemic with no change in antibiotic utilization patterns early in the pandemic among 55 community pharmacies surveyed compared with other African countries including Nigeria
Nigeria, 2023 [113]	• Randomised trial to assess if access to C-reactive protein (CRP) test kits coupled with staff training on how to use these tests improved appropriate RTI management<br>• In private community pharmacies, pharmacists can reduce non-prescription antibiotic dispensing for RTIs through appropriate interventions including greater patient education	Antibiotic dispensing decreased by 15.66% (209/300 [intervention] vs 256/300 [control]) in the adjusted analysis

NB: AMR = Antimicrobial Resistance; ARI = Acute Respiratory Infection; ASP = Antimicrobial Stewardship Program; HCPs = healthcare Professionals; PHC = Primary Healthcare; STIs = Sexually Transmitted Infections; URTIs = Upper Respiratory Tract Infections; UTIs = Urinary Tract Infections
